# Population genetics for conservation of spadefoot toads, *Pelobates fuscus*, in Western and Central Europe

**DOI:** 10.1098/rsos.240893

**Published:** 2025-07-30

**Authors:** Caroline C. Mouton, Karolin Eils, Johan Auwerx, Paolo Eusebio Bergó, Wilbert Bosman, Angelica Crottini, Julia Dayon, Christophe Eggert, Christian Göcking, Wolf-Rüdiger Große, Werner Kloas, Spartak Litvinchuk, Katarina Ljubisavljevic, Claude Miaud, Norbert Menke, Maria Ogielska, Jeroen Speybroeck, Loïc van Doorn, Richard Struijk, Matthias Stöck, Joachim Mergeay

**Affiliations:** ^1^ Research Institute for Nature and Forest, 9500 Geraardsbergen, Belgium; ^2^ Department 4, Fish Biology, Fisheries and Aquaculture, Leibniz Institute of Freshwater Ecology and Inland Fisheries in the Forschungsverbund Berlin e.V., Berlin 12587, Germany; ^3^ Naturparkverwaltung 'Hoher Fläming' im Landesamt für Umwelt, 14823 Rabenstein OT Raben, Germany; ^4^ Research Institute for Nature and Forest, 1630 Linkebeek, Belgium; ^5^ ELEADE Società Cooperativa a.r.l., 10010 Chiaverano, Italy; ^6^ Reptile, Amphibian & Fish Conservation Netherlands (RAVON), 6501 BK Nijmegen, The Netherlands; ^7^ CIBIO, Centro de Investigação em Biodiversidade e Recursos Genéticos, InBIO Laboratório Associado, Campus de Vairão, Universidade do Porto, 4485-661 Vairão, Portugal; ^8^ Departamento de Biologia, Faculdade de Ciências, Universidade do Porto, rua do Campo Alegre s/n, 4169-007 Porto, Portugal; ^9^ BIOPOLIS Program in Genomics, Biodiversity and Land Planning, CIBIO, Campus de Vairão, 4485-661 Vairão, Portugal; ^10^ CEFE, Univ Montpellier, CNRS, EPHE-PSL University, IRD, 34293 Montpellier, France; ^11^ Société Herpétologique de France and Fauna Consult, 22410 Saint-Quay-Portrieux, France; ^12^ Naturschutzbund, NABU-Naturschutzstation Münsterland eV, 48165 Münster, Germany; ^13^ Martin-Luther-Universität Halle-Wittenberg, Zentralmagazin Naturwissenschaftliche Sammlungen, 06099 Halle, Germany; ^14^ Institute of Cytology, Russian Academy of Sciences, St Petersburg 194064, Russian Federation; ^15^ Institute for Biological Research ‘Siniša Stanković’, National Institute of the Republic of Serbia, University of Belgrade, Beograd, Serbia; ^16^ Department of Evolutionary Biology and Conservation of Vertebrates, University of Wrocław, Wroclaw, Poland

**Keywords:** amphibian conservation, European common spadefoot toad, microsatellites, population genetics, *Pelobates fuscus*

## Abstract

European amphibians such as the common spadefoot toad (*Pelobates fuscus* (Laurenti, 1768)) exhibit alarming declines across large parts of their range, despite extensive legislation aimed at conservation. As knowledge about the genetic background of a species has become crucial for conservation assessment, we investigated nuclear genetic diversity and structure of *P. fuscus*, based on microsatellite loci. Clustering revealed major groups corresponding to postglacial colonization patterns. Additionally, we found contrasting patterns of genetic diversity across the 66 studied populations, reflecting the conservation status of the species in Europe and with marked genetic impoverishment across most populations in the northwestern part of the species’ distribution range. Our findings provide genetic information on the conservation status of the common spadefoot toad across Europe, emphasizing the need for urgent conservation efforts. We recommend further genetic monitoring, habitat restoration and potential translocations to safeguard the species in the face of ongoing challenges.

## 1. Introduction

Globally, amphibians face many threats, such as infectious diseases, habitat alteration and loss, industrial agriculture involving pesticides, urbanization and industrialization, environmental change and pollution, invasive species (predators, competitors and pathogens) and climate change [1,2]. However, it is the cumulative impact of these various stressors [3,4], some of which remain poorly understood, that is considered the primary driver behind the massive declines observed in amphibian populations [2].

Anthropogenic population declines can cause the onset of an extinction vortex, whereby inbreeding reduces fitness and genetic drift depletes evolutionary potential [5]. This ultimately compromises both short-term and long-term viability of populations [6,7]. Exposito-Alonso *et al.* [8] highlight that habitat loss is causing a silent genetic mass extinction, with genetic diversity losses in threatened species amounting to 20%. Even though a large body of legislation is in place to counter population declines and protect amphibians (e.g. the Bern Convention and the European Habitats Directive), European amphibian conservation has not managed to curb the negative trends. The complex interplay of various factors, including harmful anthropogenic impacts, a generally limited dispersal ability and frequent association with fragile habitats such as wetlands, exacerbates the vulnerability of amphibians [9]. Consequently, populations of many amphibian species have become highly susceptible to the loss of genetic diversity [9] and local extinctions [2].

Recognizing the importance of genetic diversity for population viability, understanding the genetic background and structure of threatened species has become a vital part of species’ conservation [7,10,11]. This study focuses on the common spadefoot toad (*Pelobates fuscus* (Laurenti, 1768)), a wide-ranging fossorial species distributed across lowland Central and Eastern Europe. The Western Palearctic genus *Pelobates* (Wagler, 1830) comprises Iberian *P. cultripes* (Cuvier, 1829), northwest African *P. varaldii* (Pasteur & Bons, 1959), Central to Eastern European and north Italian *P. fuscus*, Eastern European *P. vespertinus* (Pallas, 1771), Asia Minor *P. syriacus* (Boettger, 1889) and the recently resurrected *P. balcanicus* (Karaman, 1928) in the Balkans [12]. The north Italian *P. fuscus* populations were long considered a subspecies (*P. f. insubricus*) but Litvinchuk *et al.* [13] synonymized this taxon with *P. fuscus*. Although its global conservation status is listed as ‘least concern’ on the European Red List [14], the species is in steep decline across many parts of its range [15]. The conservation status under the EU Habitats Directive is only considered ‘good’ in 3 out of 18 countries, while it remains ‘unknown’ in 2, ‘poor’ in 5 and ‘bad’ in 8 countries [16]. Earlier genetic studies on the common spadefoot toad focused on phylogeographical analyses [13,17–19], and most of them were limited to the analyses of mitochondrial genetic markers [13,17,18]. Eggert *et al.* [18] found low genetic divergence, suggesting recent postglacial colonization. Restricted gene flow and isolation-by-distance patterns indicated environmental changes shaped population structure. Crottini *et al.* [17] confirmed two major genetic lineages and identified three glacial refugia, emphasizing climatic influence on differentiation. The Po Valley was highlighted as a genetic diversity centre. Litvinchuk *et al.* [13] identified two main genetic groups with a narrow hybrid zone, suggesting distinct evolutionary lineages. They proposed at least four glacial refugia and evidence of postglacial range expansion. Nevertheless, while these studies had already revealed the existence of different lineages within *P. fuscus*, they offered limited insights in the conservation status of local populations. In this study, we revisited several earlier sampled populations and incorporated additional ones, analysing them using nuclear microsatellite markers. Our study focuses on patterns of nuclear genetic diversity and structure within and between populations of the common spadefoot toad, in order to ultimately assess the conservation status of populations along the western range edge.

## 2. Material and methods

### 2.1. Study area and sampling methods

**Figure 1.**
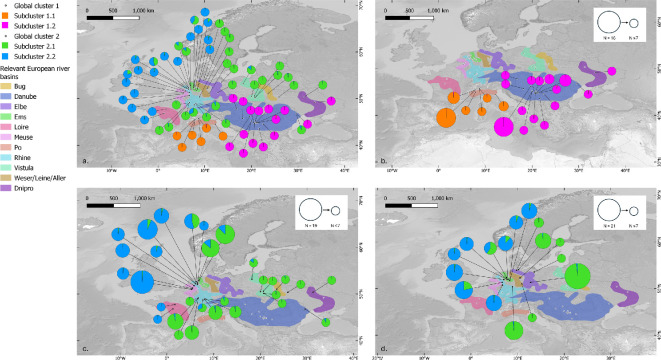
Global Bayesian STRUCTURE-clustering (Δ*K*-based) for all west European samples analysed in this study resulted in an optimum of two clusters (*K* = 2; electronic supplementary material, figures S10 and S11). Each cluster then underwent an individual STRUCTURE-analysis. An optimal number of two clusters was found for the first global cluster (pink and orange) and for the second global cluster (green and blue) as well. (a) The pink and orange locations form the first, eastern cluster; the blue and green locations form the second, western cluster. The map displays the mean assignments to each cluster (west and east) for all of the analysed locations. (b) Detailed overview of the mean assignments to the subclusters within global cluster 1, (c) detailed overview of the mean assignments to the subclusters within global cluster 2 (German samples excluded) and (d) assignment of the German samples to the subclusters within global cluster 2. Size of the pie charts in maps (b–d) corresponds with sampling size per site *N*. Alongside the pie charts depicting mean assignment per location, the relevant European river basins are also indicated. For a more comprehensive breakdown of the different clusters, see electronic supplementary material, figures S12–S15.

In this study, we used *P. fuscus* samples from 66 populations across the species’ range, with 18 of these populations previously analysed as part of an academic qualification (K. Eils, unpublished master thesis, Humboldt-Universit’t zu Berlin) (figure 1; electronic supplementary material, figure S1 and table S1). The majority of DNA samples were collected from adult specimens between 1986 and 2020, primarily through tissue samples from deceased individuals (such as toe clips and liver tissue) and buccal swabs from live adults. In addition, a smaller number of samples were obtained from freshly hatched tadpoles or embryos from clutches. For the latter, we removed full siblings from the dataset to reduce biased allele frequency estimates [20]. Tissue samples were preserved in 100% ethanol at −20°C, while cotton swabs were stored at −20°C within a few hours or days after collection. All parties involved possessed adequate sampling permits that conform with national or regional law.

### 2.2. DNA extraction, DNA amplification and microsatellite analysis

DNA from tissue samples was extracted using either a DNeasy Tissue Kit (Qiagen) or a 96 DNA Plant Kit (Qiagen, Germany) with or without the use of a BioSprint robotic workstation, according to the manufacturer’s protocols. DNA extracts of *P. fuscus* analysed in previously published and unpublished studies were also used [13,17] (S. Federici, unpublished). Total genomic DNA of these samples was extracted from tissue samples using an overnight Proteinase K digestion (concentration 10 mg ml^−1^) followed by a standard high-salt extraction method [21]. For gDNA extraction of swabs, the protocol by Broquet *et al.* [22] was used. Initially, test sequencing of the mitochondrial control region (D-loop) was carried out. The non-coding D-loop comprises 1.4 kb in spadefoot toads [23] and is expected to exhibit a higher substitution rate than cytochrome b that is barely variable in Central Europe [17]. However, presumably due to pronounced secondary structures and repetitive sections, the D-loop could not be sequenced successfully with affordable techniques and thus appeared unsuitable for further population genetic analyses. The microsatellites used in this study originated from two sources: a transcriptome made from pooled organs prepared and screened by the Leibniz Institute of Freshwater Ecology and Inland Fisheries (IGB, GenBank PRJNA357879) and a classical enriched microsatellite library, prepared by the Swiss company Ecogenics [24]. For the design at the IGB, the program Primer3 v. 0.4.0 (http://bioinfo.ut.ee/primer3-0.4.0/) was used. Only primers with a repeat motif of 4 bp and more than five repetitions were selected. Following thorough assessments of their variability (differences in lengths, similar annealing temperatures (*T*_a_) and no loops or primer dimers being formed between loci), 14 markers were selected and amplified (K. Eils, unpublished master thesis, see above). We genotyped polymerase chain reaction (PCR) amplicons (for PCR conditions, see electronic supplementary material, tables S2 and S3) on a capillary sequencer ABI 3500 or 3500xL Genetic Analyser (Applied Biosystems) and used the GeneMapper^®^ software package to score the samples.

### 2.3. Genetic data analysis

#### 2.3.1. Filtering

Before filtering, the dataset comprised 570 genotypes from 66 sampling sites, containing information for 14 loci. First, we tested for missing data, removing samples or loci with more than 20% missing data. Second, one monomorphic locus was removed. Next, we addressed possible sibling relationships. When sampling larvae from the same pond and/or clutches, a bias in allele frequencies can be introduced [20]. We, therefore, removed full siblings from the dataset. Full-sib relationships were assessed using best maximum likelihood (ML) sibship assignment with Colony v. 2.0.6.8 [25]. The following settings for this assignment were derived from Cox *et al.* [26]: polygamous males, monogamous females, inbreeding included, medium run length and three runs. Using the best (ML) sibship assignment, members of the same full-sib family except one were removed from the dataset.

In addition, we applied Genepop v. 4.3 [27] to assess possible locus-specific deviations from Hardy–Weinberg equilibrium and to explore linkage disequilibrium for each pair of loci per location, then corrected for multiple testing using the Bonferroni method [28]. A Markov chain method and default parameters were used. Decisions based on these results took also the number of samples per site into account. We also used Genepop v. 4.3 to explore the ML estimation of null allele frequency. Based on Hardy–Weinberg disequilibrium, one locus was omitted from the dataset. Finally, after filtering, 334 individual genotypes originating from the 66 populations were retained for population genetic analysis (figure 1) based on genetic information from 12 loci (Pf10635s, Pf14239s, Pf16076s, Pf9847s, Pf5754s, Pf9791sB, Pf3505s, Pf4451s, Pf8461s, Pf6194, Pf981 and Pf4582; electronic supplementary material, table S2).

#### 2.3.2. Genetic diversity

The following measures of genetic diversity were calculated for each population over all loci: mean allelic richness (AR), observed and unbiased expected heterozygosity (*H*_o_ and *uH*_e_). The genetic measures *H*_o_ and *uH*_e_ were calculated using GenAlEx 6.5 [29]. AR was calculated and adjusted for sample size (rarefaction to *N* = 5) using HP-Rare v. 1.1 [30]. The calculations of genetic diversity were based on information obtained from all 66 populations. Since samples were taken over a long period of time (35 years), we tested if there was a unique effect of sampling year on genetic diversity, which could affect downstream conclusions. For this, we performed a linear regression analysis in which we first included spatial coordinates as covariates and then added year of sampling as explanatory variable against *H*_o_.

#### 2.3.3. Genetic structure

Calculation of pairwise *F*_ST_ values (measuring fixation) and *D*_EST_ values (measuring differentiation) [31] was carried out in R v. 4.3.0 [32] using R packages DiveRsity v. 1.9.90 [33] and hierfstat v. 0.5-10 [34]. Confidence intervals of 95% are based on 1000 bootstraps. For the analysis of these indices of genetic structure, we excluded locations with sample sizes smaller than 5, thereby reducing the number of populations available for interpretation from 66 to 30. Genetic principal components analysis (PCA) ordination and the *k*-means non-parametric discriminant analysis of principal components (DAPC) clustering, including all genotypes (66 sampling sites), were performed using the adegenet v. 2.1.5 package in R [35]. We tested for isolation-by-distance (IBD) by performing a Mantel test between the genetic and the geographical distance matrices using the ade4 v. 1.7-22 package in R [36]. Next, we explored Bayesian clustering assignment through the programs Bayesian Analysis of Population Structure (BAPS) v. 6 [37] and STRUCTURE v. 2.3.4 [38]. In BAPS, we used the spatial model for mixture clustering of individuals. With *K*-values ranging from *K* = 1 to 20, ten runs per *K* were ran. Clustering analysis in STRUCTURE was performed for *K* = 1 to 20 clusters (10 runs per cluster), using an admixture ancestry model with burn-in period of 100 000 iterations and 500 000 Markov chain Monte Carlo (MCMC) replications. The best clustering option for the STRUCTURE results was determined using Structure Selector [39] and CLUMPAK [40]. Ultimately, the optimal number of clusters was compared between the Δ*K* ‘Evanno method’ [41], the ‘Puechmaille method’ [42], the results of the BAPS analysis and *k*-means analysis. For visualization of the individual membership values (*Q*_i_), based on the STRUCTURE results, we constructed barplots in R v. 4.3.0 [32] and CLUMPAK [40]; the mean population membership values (*Q*_mp_) are used for visualization in QGIS 3.28.0-Firenze [43]. Following the main STRUCTURE results, we performed subclustering in a hierarchical manner [44]. After determining the optimal number of global STRUCTURE-clusters (Δ*K*-based), each of these clusters underwent an individual clustering assignment analysis on its own to explore deeper structure within. For these analyses, parameters stayed the same as used for the initial clustering analysis. Populations with mean assignment scores around 0.50 ± 0.05 across two different clusters were subsequently included within those two clusters. For example, when a sampling location shows a mean assignment score of 0.45 to cluster A and a 0.55 assignment score to cluster B, the location will be analysed within both clusters separately. The range of explored *K* values per subcluster depends on the number of sampling sites grouped into each subcluster and ranges from *K* = 1 to *K* equals the number of populations in a studied subcluster. Finally, we checked for recent population declines using BOTTLENECK v. 1.2.02 [45] with default settings and all three mutation models. As nearly all populations have knowingly undergone population or connectivity declines, BOTTLENECK could answer largely to what extent we are able to detect these events. Six out of 66 sites (Zonhoven (BE), Muldenaue (DE), Torino Ivrea, Maceratoio Cascinette d’Ivrea (IT), Gorssel and Valthe (NL), and Hrastovača (SR)) were deemed to have sufficiently large sample sizes (*N* > 13) for bottleneck analysis.

## 3. Results

### 3.1. Genetic diversity

**Figure 2.**
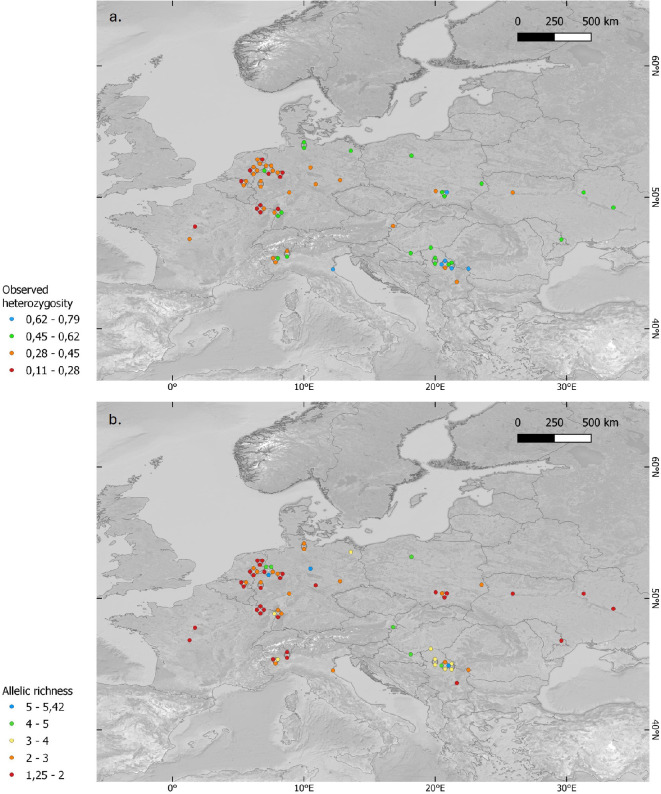
Map visualizing (a) the observed heterozygosity (*H*_o_) based on 12 microsatellite loci across the studied range. *H*_o_ ranges from 0.111 to 0.792. An east-to-west gradient can be observed where the highest values are found in the east and correspond to known refugia of *Pelobates fuscus*. (b) Results of the calculated allelic richness (*AR*; adjusted for a sample size of 5) per sampling location. Measures of allelic richness are based on a set of 12 microsatellite markers.

Average allelic richness per locus (*AR*) (rarefaction to *N* = 5) ranged from 1.25 to 5.42 per population, with the lowest value observed in Lorraine carrière 3 maisons (FR) and the highest value observed in Braunschweig (DE) (figure 2; table 1). Sample sizes in many Eastern European populations prohibited a robust assessment of *AR* and *uH*_e_ there. Gene diversity (*uH*_e_; unbiased expected heterozygosity) was the lowest in France (Lorraine Carrière Merle, Carrière Saint Avold; *uH*_e_ = 0.106 ± 0.058) and the highest in Poland (Pojawie, Lasy Radłowskie; *uH*_e_ = 0.75 ± 0.13) (table 1). Observed heterozygosity (*H*_o_; average value per individual across loci) ranged between 0.11 ± 0.06 (Lorraine Carrière Merle, Carrière St-Avold (FR)) and 0.79 ± 0.10 (Balta Iakuri (RS)) (figure 2; table 1). Year of sampling did not significantly affect genetic diversity (*H*_o_) after taking latitude and longitude into account. Longitude, however, showed a clear decrease from west to east (electronic supplementary material, figures S2 and S3). While both western and eastern populations showed similar upper values around 0.5, severely depleted populations (*H*_o_ < 0.35) were only detected in Western European populations, with the most pronounced levels of low diversity observed in the Grand-Est region of northeastern France (Lorraine region; 0.11 < *H*_o_ < 0.31).

**Table 1.** Overview of the populations (with sample sizes) analysed and their corresponding genetic statistics. Number of genotyped individuals (*N*), allelic richness (AR; rarefaction to *N* = 5), mean observed heterozygosity (*H*_o_) over all loci and corresponding standard errors (s.e.), and mean unbiased expected heterozygosity (*uH*_e_) over all loci and corresponding s.e. Sampling sites with less than five samples per sampling location are indicated in italic.

sampling locations	location code	*N*	AR (adjusted)	*H*_o_ ± s.e. (mean over loci)	*uH*_e_ ± s.e. (mean over loci)
**Austria**					
*Neusiedlersee*	*AT_NEUS*	*3*	*4.25*	*0.361 ± 0.096*	*0.439 ± 0.107*
**Belgium**					
Peer	BE_PEER	5	1.92	0.333 ± 0.093	0.293 ± 0.075
Zonhoven	BE_ZON	19	2.01	0.32 ± 0.079	0.354 ± 0.082
**Croatia**					
*Donji Miholjac*	*HR_DONJI*	*2*	*4.17*	*0.458 ± 0.114*	*0.528 ± 0.106*
**France**					
*Alsace Brumath*	*FR_ALSACE_Bru*	*2*	*1.75*	*0.458 ± 0.13*	*0.417 ± 0.113*
*Alsace Mothern*	*FR_ALSACE_Moth*	*2*	*2.08*	*0.5 ± 0.123*	*0.569 ± 0.113*
Alsace Roppenheim	FR_ALSACE_Ropp	7	2.12	0.262 ± 0.06	0.33 ± 0.077
Alsace Sauer	FR_ALSACE_Sau	5	3.08	0.375 ± 0.082	0.558 ± 0.079
*Indre*	*FR_INDR*	*2*	*1.58*	*0.375 ± 0.109*	*0.319 ± 0.083*
*Loiret*	*FR_LOIR*	*2*	*1.42*	*0.25 ± 0.097*	*0.222 ± 0.08*
*Lorraine carrière 3 maisons*	*FR_LOR_3Maisons*	*2*	*1.25*	*0.167 ± 0.094*	*0.139 ± 0.073*
Lorraine N2000	FR_LOR_N2000	9	1.85	0.306 ± 0.11	0.264 ± 0.091
*Lorraine Carrière Merle, Carrière Saint Avold*	*FR_LOR_St_Avold*	*3*	*1.33*	*0.111 ± 0.063*	*0.106 ± 0.058*
Lorraine Zang	FR_LOR_Z	7	1.88	0.214 ± 0.08	0.234 ± 0.085
**Germany**					
*Bad Ramstedt*	*DE_BAD_RAM*	*3*	*2.17*	*0.528 ± 0.119*	*0.45 ± 0.088*
Bingenheimer_Ried	DE_BING	11	2.05	0.363 ± 0.075	0.376 ± 0.071
*Braunschweig*	*DE_BRAUN*	*1*	*5.42*	*0.333 ± 0.142*	*0.333 ± 0.142*
Erfstadt-Scheuren	DE_ERFSTADT	8	1.96	0.354 ± 0.097	0.344 ± 0.082
*Erfurt*	*DE_ERFURT*	*1*	*1.33*	*0.333 ± 0.142*	*0.333 ± 0.142*
Fürstenkuhle_altwasser	DE_FUR	9	5.1	0.278 ± 0.079	0.285 ± 0.065
Heiliges Meer	DE_HEI	5	2.42	0.363 ± 0.079	0.428 ± 0.074
Heidbergmühle	DE_HEID	10	2.24	0.385 ± 0.074	0.447 ± 0.067
*Holzendorf*	*DE_HOLZ*	*1*	*3.42*	*0.5 ± 0.151*	*0.5 ± 0.151*
Rote Beeke, Lipstadt	DE_LIPSTADT	6	1.79	0.156 ± 0.063	0.252 ± 0.064
MB_12	DE_MB_12	5	4.83	0.317 ± 0.087	0.25 ± 0.066
MB_13	DE_MB_13	6	4.9	0.347 ± 0.095	0.269 ± 0.067
MB_Ls	DE_MB_Ls	8	1.86	0.231 ± 0.061	0.287 ± 0.06
Muldenaue	DE_MULD	21	2.57	0.369 ± 0.068	0.46 ± 0.069
Torfvenn	DE_TORF	7	2.23	0.399 ± 0.059	0.421 ± 0.065
Schleswig Holstein, Trappenkamp	DE_TRAP	8	2.45	0.458 ± 0.083	0.424 ± 0.079
**Italy**					
*Ravenna*	*IT_RAV*	*4*	*2.42*	*0.625 ± 0.125*	*0.509 ± 0.094*
Torino Ivrea, Maceratoio Cascinette d'Ivrea	IT_TOR_IVR_MAC	16	2.83	0.318 ± 0.061	0.471 ± 0.083
Torino Ivrea, Torre Canavese, Stagno San Giacomo	IT_TOR_IVR_TORRE	6	3.25	0.458 ± 0.104	0.511 ± 0.101
*Torino Poirino, Cascina Bellezza*	*IT_TOR_POI*	*1*	*1.33*	*0.333 ± 0.142*	*0.333 ± 0.142*
*Varese Arsago Seprio, acquitrino Monte Brano*	*IT_VAR_Arsago_Seprio*	*1*	*1.5*	*0.5 ± 0.151*	*0.5 ± 0.151*
*Varese Somma Lombardo, Acquitrino a goccia*	*IT_VAR_Somma_Lombardo*	*1*	*1.42*	*0.417 ± 0.149*	*0.417 ± 0.149*
**Moldova**					
*Kitskany*	*MD_PRID*	*1*	*1.5*	*0.5 ± 0.151*	*0.5 ± 0.151*
**The Netherlands**					
*Barvoorde*	*NL_BAR*	*3*	*1.67*	*0.5 ± 0.126*	*0.344 ± 0.078*
De Poll	NL_DEP	5	1.92	0.35 ± 0.074	0.335 ± 0.073
Gorssel	NL_GOR	14	2.2	0.448 ± 0.054	0.452 ± 0.04
Hendriksveen	NL_HENDR	11	1.86	0.311 ± 0.054	0.313 ± 0.071
Overasselte and Hatert fens	NL_OVER	6	2.28	0.422 ± 0.088	0.416 ± 0.072
Staphorst	NL_STAP	8	1.62	0.235 ± 0.079	0.244 ± 0.084
Strijper_Aa	NL_STR_Aa	7	1.56	0.228 ± 0.071	0.21 ± 0.061
Valthe	NL_VAL	13	1.99	0.314 ± 0.072	0.32 ± 0.061
Zieuwent	NL_ZIEUW	8	1.65	0.146 ± 0.046	0.188 ± 0.057
**Poland**					
*Bydgoszcz*	*PL_BYD*	*1*	*4.75*	*0.500 ± 0.151*	*0.500 ± 0.151*
*Close to Chełm*	*PL_CHELM*	*2*	*2.33*	*0.583 ± 0.12*	*0.583 ± 0.095*
*Dołega, Lasy Radłowskie*	*PL_DOLEGA*	*1*	*1.58*	*0.583 ± 0.149*	*0.583 ± 0.149*
*Miechów*	*PL_MIE*	*2*	*1.67*	*0.333 ± 0.112*	*0.306 ± 0.098*
*Pojawie, Lasy Radłowskie*	*PL_POJA*	*1*	*1.75*	*0.750 ± 0.131*	*0.750 ± 0.131*
*Stawiany*	*PL_STAW*	*2*	*2.17*	*0.500 ± 0.123*	*0.486 ± 0.113*
**Serbia**					
*Aleksinac-Cicina*	*RS_ALEK*	*1*	*1.42*	*0.417 ± 0.149*	*0.417 ± 0.149*
*Balta lakuri*	*RS_BALTA*	*2*	*2.58*	*0.792 ± 0.096*	*0.708 ± 0.085*
Deliblato sand, Utrine	RS_DELI	6	3.89	0.653 ± 0.088	0.598 ± 0.08
*Dubovacki rit*	*RS_DUBO*	*1*	*3.5*	*0.583 ± 0.149*	*0.583 ± 0.149*
Hrastovača	RS_HRAS	16	3.58	0.497 ± 0.064	0.6 ± 0.056
*Ivanovo*	*RS_IVAN*	*1*	*3.33*	*0.417 ± 0.149*	*0.417 ± 0.149*
*Karlovacki vinogradi*	*RS_KARL*	*1*	*3.42*	*0.500 ± 0.151*	*0.5 ± 0.151*
*Kovin*	*RS_KOV*	*4*	*5.25*	*0.521 ± 0.109*	*0.512 ± 0.102*
*Kraljevac, Obzovik bara*	*RS_KRAL*	*1*	*3.5*	*0.583 ± 0.149*	*0.583 ± 0.149*
*Krnjaca*	*RS_KRN*	*2*	*2.42*	*0.625 ± 0.109*	*0.611 ± 0.095*
*Samos*	*RS_SAMOS*	*2*	*4.67*	*0.667 ± 0.094*	*0.708 ± 0.082*
**Ukraine**					
*Novaya Troyanda*	*UA_KIEV_Nov_Troy*	*1*	*1.5*	*0.5 ± 0.151*	*0.5 ± 0.151*
*Zapselye*	*UA_POL*	*1*	*1.58*	*0.583 ± 0.149*	*0.583 ± 0.149*
*Zbitin*	*UA_ROVNO*	*1*	*1.42*	*0.417 ± 0.149*	*0.417 ± 0.149*

### 3.2. Genetic structure

The strongest differentiation was found between Italian populations on the one hand, and Belgian, Dutch and German populations on the other hand. Pairwise *F*_ST_ values ranged from 0 (BC 95% CI: 0.00–0.12) to 0.65 (BC 95% CI: 0.61 and 0.70). Pairwise differentiation (*D*_*EST*_) ranged from 0 (BC 95% CI: 0.00–0.00) to nearly complete differentiation at 0.94 (BC 95% CI: 0.89–0.99) (electronic supplementary material, figure S4 and table S4). Due to limited available sample sizes, the results of the pairwise structure values were based on 30 out of the 66 populations only. The PCA biplot mainly separates Italian and Eastern European populations from Western European populations along PC1. A Mantel test showed clear spatial genetic structure across Europe (rM = 0.507, *p* ≤ 0.0001).

**Figure 3.**
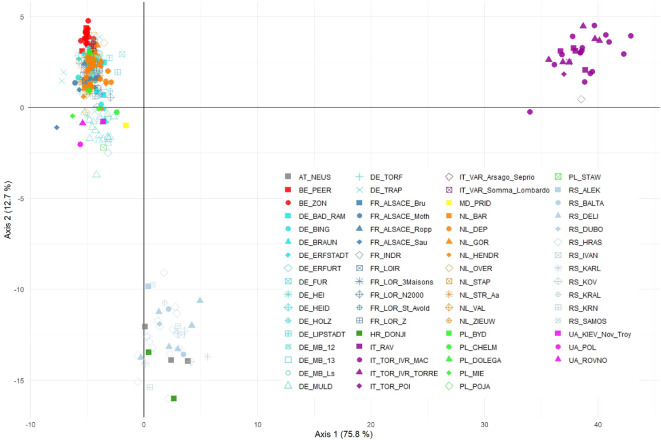
DAPC clustering where each symbol represents an individual sample from analysed sampling locations. Percentages of 75.8% and 12.7% of the variation can be explained by the first and second axis respectively. A more detailed visualization of the left-hand side, where the northwestern European sampling locations cluster together, can be found in electronic supplementary material, figure S8.

**Figure 4.**
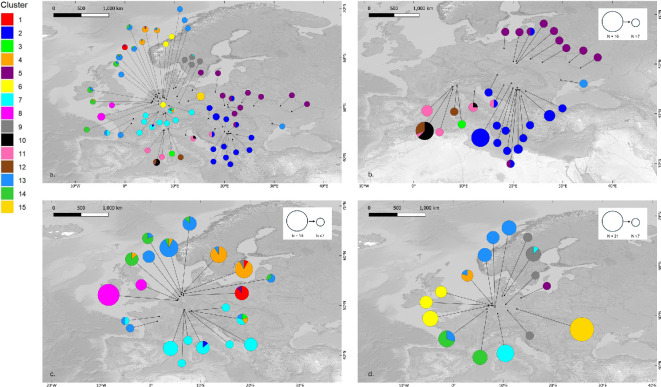
(a) Results of the BAPS spatial mixture analysis with an optimal number of clusters at *K* = 15. Although this largely mirrors STRUCTURE results, BAPS results show deeper regional genetic structure, which may be caused by stronger genetic drift. (d) Detailed overview of the studied locations in Germany (DE), (c) detailed overview of the studied locations in The Netherlands (NL), France (FR) and Belgium (BE), and (b) zoom in on the sites located more east (Austria (AT), Italy (IT), Poland (PL), Moldova (MD), Croatia (HR), Serbia (RS) and Ukraine (UA)). Size of the pie charts corresponds with sample size per site *N*.

DAPC clustering revealed an optimum at *K* = 10, based on the Bayesian information criterion (BIC) versus number of clusters plot (electronic supplementary material, figure S7). About 75.8% of the variance can be explained by the first axis and 12.7% by the second axis. The DAPC plot (figure 3) shows a pronounced division between the Italian samples, the southeastern European samples (Austrian samples included) and the rest of the European individuals. When zooming in on the latter, a subdivision in between these northwestern European samples is revealed (electronic supplementary material, figure S8). Using the Puechmaille method, an optimal number of clusters should be found at *K* = 17 (electronic supplementary material, figure S10). BAPS spatial mixture analysis concludes an optimal number of clusters at *K* = 15 (figure 4; electronic supplementary material, figure S16).

Based on the STRUCTURE results and Δ*K*, the optimal global number of clusters was 2 (figure 1; electronic supplementary material, figure S10). The first cluster (‘eastern’) predominantly encompasses Italy and southeastern Europe (including Austrian samples). The second cluster (‘western’) groups the rest of the European samples. Each of these two main clusters was then further analysed individually through a hierarchical analysis using STRUCTURE v. 2.3.4 [38] . In the global assignment (*K* = 2), the Moldovan sample from Kitskany (*N* = 1) displayed intermediate assignment scores to both clusters (*Q*_i,cluster1_ = 0.51 and *Q*_i,cluster2_ = 0.49; electronic supplementary material, figure S11). This population/sample was, therefore, included in both in-depth analyses. In what follows, we will use ‘*N*’ for the number of individuals and ‘*n*’ for the number of populations.

When analysed individually, the eastern cluster (*N* = 73, *n* = 21 and *K* = 1 to 21; electronic supplementary material, figures S12 and S13) demonstrated an optimal number of two subclusters (*K* = 2; figure 1b), separating the Italian common spadefoot toads from those in the Danube catchment and one Ukrainian location (Zapselye).

The western cluster (*N* = 262, *n* = 46 and *K* = 1 to 46; electronic supplementary material, figures S14 and S15) also showed an optimal grouping at two clusters (*K* = 2; figure 1c,d). The division into two clusters segregated the samples from the northern Netherlands, along with the German toad population (Ems basin and Rhine basin excluded) and the northeastern French samples from the Rhine basin into a first subcluster. Some samples displayed ambiguous assignments, the Zieuwent (NL) and Heiliges Meer (DE). Finally, the central French samples and the Belgian samples were grouped together with the remaining German samples from the Rhine and Ems basin. The global STRUCTURE results for other *K*-values are provided in the electronic supplementary material, figure S9. The BAPS mixture results (optimal *K* = 15) reveal more regionality and visualize the substructuring within the western and eastern clusters individually roughly defined by STRUCTURE. Finally, for three out of six analysed sites (Zonhoven (BE), Muldenaue (DE) and Gorssel (NL)), the bottleneck results reveal a shifted mode (electronic supplementary material, figures S17 and S18), indicating that these populations probably went through a recent bottleneck event.

## 4. Discussion

We present a comprehensive overview of nuclear genetic diversity and population genetic structure across a significant portion of the western range (66 locations) of the common spadefoot toad, *P. fuscus*. Although samples had been collected across a long period of time, this had no effect on genetic diversity estimates after correcting for sampling location. Despite some populations being represented by only a few or even a single genotype, our results allow general inferences regarding genetic status to be used to inform conservation. In this section, we first shed light on the genetic diversity of common spadefoot toads across its studied locations. Next, we elaborate on our findings concerning the population structure.

Previous studies focusing on the common spadefoot have indicated a complex phylogeographic history, characterized by distinct regional clusters that have undergone largely separate evolutionary trajectories since the late Pleistocene [13,17] and which currently have disjunct distributions. This phylogeographic history largely explains the nearly complete genetic differentiation among populations across regions (e.g. Italian versus Dutch and Belgian populations). We also find an east-to-west decline in genetic diversity, which likely reflects sequential founder events during postglacial recolonization [13,17,46]. Similar phylogeographic patterns and differentiation levels have been found in other European amphibian species, where sequential founder events from glacial refugia and genetic drift have shaped large-scale gradients in genetic diversity (e.g. [47,48]), with negative consequences for fitness in populations at the edge of the distribution (reviewed in [48]). When focusing on the populations with minimum sample sizes of 5, we consistently observed lower levels of gene diversity (*uH*_e_) in Western European countries. Additionally, patterns of *AR* (adjusted to a sample size of 5) demonstrated a similar association with the local and regional conservation status of common spadefoot toads (figure 2; electronic supplementary material, figure S19). While the phylogeographic history may partially account for lower levels of genetic diversity in western populations, many populations exhibited reduced diversity levels compared with nearby populations with the same phylogeographical origin. This decline in genetic diversity is likely attributable to strong genetic drift due to recent population decline [49]. Spadefoot toads are often associated with open environments with high levels of natural disturbance on loose (sandy) soils, such as river floodplains and dune slacks. In Western Europe, natural floodplain dynamics have been largely lost due to human river management and changed human landscape use, and spadefoot toads found a surrogate secondary habitat in man-made ponds in extensive agricultural landscapes (e.g. [50]). Following agricultural intensification since the 1950s, this secondary habitat has been lost increasingly in Western and Central Europe (e.g. [51]), whereas primary and secondary habitats, though declining too, are still present in parts of Eastern Europe (reviewed in [18]). This loss of metapopulation structure has increased genetic drift and is likely contributing to a genetic extinction spiral. At present, most of the Western European populations are extremely fragmented and should likely be considered as isolated remnants experiencing no gene flow with other populations anymore.

Irrespective of geographical position within the studied range, we were able to observe populations with high levels of genetic diversity (*uH*_e_ or *H*_o_ > 0.5; electronic supplementary material, figure S2). Nonetheless, we note an apparent decline in genetic diversity showing an east-to-west-gradient. Official reports to the EU Habitats Directive (Council Directive 92/43/EEC) indicate a ‘bad’ to ‘poor’ conservation status of spadefoot toads across Austria, Belgium, France, Germany, Italy, The Netherlands and Poland (electronic supplementary material, figures S19) [52]. Our population-specific genetic diversity levels confirm this assessment. Within each of these countries, a significant proportion of the populations exhibited reduced levels of genetic diversity. We applied a threshold of observed heterozygosity (*H*_o_) lower than 0.4 to denote genetic depletion, indicating a loss of 20% or more relative to non-impoverished populations in the same region. Following this definition, all Austrian and Belgian spadefoot toad populations are classified as genetically impoverished, along with 12 out of 16 German *P. fuscus* populations, 8 out of 10 French populations, 6 out of 9 Dutch populations, 2 out of 6 Italian populations and 1 out of 6 Polish populations. In contrast, none of the populations from Croatia, Moldova, Serbia or Ukraine meet this definition of genetic depletion. We did not report inbreeding (FIS) as this coefficient reflects to what extent there is a deviation from Hardy–Weinberg expectations, which may or may not be caused by a lack of mate choice (due to small census size (*N*_c_), but which could also represent unnoticed spatial genetic structure); nor did we report estimates for effective population size (linkage disequilibrium based *N*_e_; *LDN*_e_) due to these estimates being extremely sensitive to model assumptions such as random sampling, random mating within the populations etc. (e.g. [53,54]). Next, three out of six locations fit for BOTTLENECK analysis showed a shift from the ‘normal’ L-shaped distribution to a more uniform distribution. This shift can occur when, due to for example a bottleneck event, rare alleles become less frequent in the population. A bottleneck is not merely a reflection of a change in local effective size (*N*_e_), but also in gene flow, as a reduction in gene flow changes the realized inbreeding effective size of a subpopulation in a formerly connected metapopulation [55]. However, given that all European populations have experienced declines, not finding a bottleneck via the BOTTLENECK software is not a proof that no bottleneck occurred.

Across regions, we observe relatively clear large-scale phylogeographical signals, indicating postglacial connections between the Italian populations from the Po River basin and the Danube valley, as previously suggested by Crottini *et al*. [17]. The distinction of the Italian samples was quite clear in the DAPC analysis. Contrary to this, it is only when we impose a requirement for 11 or more clusters in STRUCTURE (electronic supplementary material, figure S10) that we identify a distinct cluster for the Italian samples. Whether or not the Italian populations should be considered a distinct subspecies (see [13]) is largely a matter of systematics opinion; importantly, the Italian populations belong to a genetically distinct and geographically discrete lineage that has long been isolated from other populations. As such, it is a relevant conservation unit that should be maintained, irrespective of a potential subspecies status. STRUCTURE, designed to minimize both within-groups linkage disequilibrium and Hardy–Weinberg disequilibrium, has little statistical power to assign few orphan samples to biologically meaningful groups. This also explains why some samples (e.g. from Moldova) do not seem to fit particularly well in any of the groups. Conversely, when sample sizes are very large (e.g. the German Muldenaue samples or the Belgian samples), Bayesian clustering has more power to detect meaningful patterns and finds additional clusters. As clustering algorithms are ignorant of the underlying processes creating the patterns they provide, this does not necessarily mean they have a different phylogeographical history or are truly genetically more distinct. Following the STRUCTURE genetic clustering analysis, the optimal number of clusters was determined to be two (Δ*K*-based), dividing the Western European common spadefoot toad populations into (i) an Italian/Pannonian/western Balkan group in the south and (ii) a cluster containing individuals from northeastern France (Alsace-Lorraine), the northern part of The Netherlands and all samples from Germany together with the Belgian and Dutch spadefoot toads. Exploring shallower genetic structure within these clusters revealed further substructuring, which was already largely visible in earlier studies of the mitochondrial phylogeography of the species [17,18]. Focusing on the Atlantic Rhine region, common spadefoot toad populations from the middle Rhine catchment all cluster together, while populations from the lower Rhine Valley align with neighbouring populations from the Meuse catchment in Belgium and The Netherlands. A gradual transition is observed from the Rhine towards the Ems valley across the Dutch plains. The Ems valley marks the beginning of a transition between the subclusters of global cluster 2, which continues eastward until it reaches the Weser/Leine/Aller river system, where the split is fully realized. BAPS clustering (figure 4) shows a more regional genetic structuring, which may be caused by stronger genetic drift. Although more clusters are defined using BAPS, these results still largely mirror the results found with STRUCTURE. The BAPS results are in line with the known poor connectivity between populations and confirm the IBD results. Spadefoot toads very rarely disperse further than 2000 m from their natal site [56,57], making them very sensitive to habitat fragmentation and a loss of gene flow. At least in France [58], Belgium [59] and The Netherlands [60,61] nearly all populations have become functionally isolated from other populations, making them very vulnerable to further losses of genetic diversity.

It is thought that *P. fuscus* found refuge during the Last Glacial Maximum in the Pannonian Basin around the Sava River (tributary of the Danube) and the Po Plain south of the Alps [13,17,62]. From these refugia, common spadefoot toads probably recolonized northern and Western Europe during the Holocene [13]. As found for *Triturus* newts, possible other additional refugia could be presumed in southern Germany and northeastern France according to the Model for Interdisciplinary Research on Climate distributional model [63]. We detected the highest observed heterozygosity (*H*_o_) across southern European populations, with the population from Torino Ivrea being a notable exception (*H*_o_ = 0.318; figure 2). This phylogeographical history of glacial refugia and postglacial founder events likely left their marks in the form of somewhat lower nuclear genetic diversity in Western European populations (as reflected by mitochondrial diversity [17]), but this alone cannot explain the large variance in genetic diversity across Western European populations, and we propose that local depletion of genetic diversity is rather caused by recent population declines and strong genetic drift associated with it. Across the study region, we observed the entire range of genetic differentiation among populations, up to nearly total separation (*D*_EST_ > 0.9). Thus, this likely reflects a combination of ancient phylogeographic differentiation [17], and recent genetic drift in small populations leading further to a path of reciprocal fixation/loss of alleles. The high *F*_ST_ and *D*_EST_ results are also reflected in the DAPC clustering (figure 3), where the Italian samples separate themselves from (i) the north-western European samples and (ii) Serbian, Croatian and Austrian samples.

Finally, the isolated populations in central France have long posed a conundrum: are these populations ancient relics or were they introduced? Amphibians have often been transported and introduced deliberately or accidentally across Europe: examples abound for water frogs (*Pelophylax* sp.) (e.g. [64]), African clawed frogs (*Xenopus laevis*) (e.g. [65,66]), Italian crested newts (*Triturus carnifex*) [67], Alpine newts (*Ichthyosaura alpestris*) (e.g. [68,69]), tree frogs (*Hyla* sp.) [70] and American bullfrogs (*Lithobates catesbeianus*) (e.g. [71,72]) but also common spadefoot toads (*Pelobates fuscus*) [73]. Two populations of *P. fuscus* from central France (Indre (*N* = 2) and Loiret (*N* = 2), Loire Basin) fall well outside of the present distribution of the species. However, various natural history reports from the nineteenth century mention the presence of *P. fuscus* across the Loire and the Seine Basins (e.g. [74] in [75]; [76]). The species may well have had a wide but scant distribution across northern France, with only few populations remaining. Whether or not these populations are true relicts, or whether they represent recent introductions may seem academic at first glance, but it makes a great difference how policy considers the need to conserve these populations. BAPS structuring shows that half of the Loiret (*N* = 2) samples do cluster together with the northeastern French samples (figure 4; electronic supplementary material, figure S16). This pattern may as well be explained by a common phylogeographic ancestry, just like Ukrainian samples being grouped with samples from northern Germany (Holzendorf; electronic supplementary material, figure S16). Contrary to this, based on the STRUCTURE analysis of the second global cluster (figure 1), samples from these central French populations clustered with populations from Belgium, The Netherlands and Germany rather than with the cluster containing the populations from the middle Rhine in northeastern France. This, however, does not necessarily support a recent introduction for these populations. Determining the ‘true’ origin of these populations will require larger sample sizes (for this study, only four samples were available for structure analysis), more genetic markers and explicit testing of alternative demographic scenarios. Given the historical presence of this species in the region, further conservation and active management of these populations are warranted.

## 5. Conclusion

Despite the limited sampling, clear patterns of within-population genetic diversity were detected. We showed depleted levels of genetic diversity across many populations in the western, central and northern part of the *P. fuscus* distribution range. While this may be partly due to historical Holocene colonization, it likely also results from genetic drift in declining populations. These patterns indicate that the conservation genetics status of many common spadefoot toad populations is concerning and, especially in its northwestern range, requires urgent attention and intervention. With alarming decline trends in many countries of the European Union [52], we encourage that further action should be taken to ensure the survival of this species in Europe, including adequate monitoring of the species’ genetic diversity and appropriate conservation listings and measurements under national legislation. Currently, several action plans and conservation translocation projects are ongoing in Europe, including reintroduction of the species in favourable habitat and genetic rescue in genetically impoverished populations, following established guidelines for conservation translocations [77,78]. Examples of such actions can be found in, for example, Belgium [59], The Netherlands (e.g. [79,80]), Italy (e.g. [81]) and Germany (LIFE11 NAT/DE/000348 [82]). Finally, we underline the importance of the conservation, restoration, maintenance and creation of relevant natural and semi-natural habitats as a key factor for the persistence of common spadefoots in Western Europe [62,83].

## Data Availability

Data are available via Dryad [84]. Electronic supplementary material is available online [85].
